# Preparing Europe for future health threats and crises: the European Health Union

**DOI:** 10.2807/1560-7917.ES.2023.28.5.2300066

**Published:** 2023-02-02

**Authors:** Sandra Gallina

**Affiliations:** 1 Directorate-General for Health and Food Safety, European Commission, Brussels, Belgium

**Keywords:** public health, Europe, health threats, crisis, pandemic

It has been a little over 3 years since a novel coronavirus, the severe acute respiratory syndrome coronavirus 2 (SARS-CoV-2), spread across Europe and the world. During that time, health systems were put under pressure like never before. The coronavirus disease (COVID-19) pandemic revealed that most health systems were ill-equipped for a crisis disproportionately affecting the most vulnerable communities. The pandemic also uncovered the persistent inequality in prevention and care between and within countries in the European Union (EU) and between different population groups. Economies and societies continue to face uphill battles in coping with the lingering effects of a disease that led to too many lives lost.

Faced with an unprecedented challenge, the European Union (EU) took action; for example, during the COVID-19 pandemic, closer coordination and collaboration at EU level, with and between countries, resulted in the EU Vaccines Strategy [1] and the EU COVID-19 digital certificate [2]. Moreover, recognising the limitations of the early response to the pandemic at EU level, the European Commission (EC) presented a set of proposals in November 2020, to substantially strengthen health security by creating a strong European Health Union.

The aim of the Health Union is to reinforce coordination at EU level to build healthier, more resilient and more sustainable societies for the future. The building blocks of this Health Union are steadily being put into place with major developments achieved in the last year. These include a revised and stronger mandate for the European Centre for Disease Prevention and Control (ECDC) [3] and the European Medicines Agency (EMA) [4], the new Health Emergency Preparedness and Response Authority (HERA) [5], the Pharmaceutical Strategy for Europe [6], Europe’s Beating Cancer Plan [7], the European Health Data Space [8] and the Global Health Strategy [9].

To shield present and future generations against the worst effects of future health threats, it is important to be properly equipped to face health challenges. This starts by relying on fit for purpose legislation that can affect real change. The Decision No 1082/2013/EU on serious cross-border threats to health [10] has thus far worked well but in a post-pandemic world, a limited legal framework will no longer do. The Health Union package entails a new overarching framework for health security: a Regulation on serious-cross border threats to health that entered into force on 26 December 2022 [11]. This Regulation aims to ensure a robust preparedness planning, a more integrated surveillance system and a better capacity for accurate risk assessment and targeted response. It allows to set up solid mechanisms for joint procurement of medical countermeasures and includes the possibility to adopt common measures at the EU level to address future cross-border health threats. The Regulation also strengthens the role of the Health Security Committee (HSC) as coordinating body of the EU level response. The HSC assumes additional responsibilities in the adoption of guidance and opinions to better support countries in the prevention and control of serious cross-border threats to health. To further promote an effective and coordinated EU response, the EC, in cooperation with countries and the relevant EU agencies, will develop an EU Prevention, Preparedness and Response Plan and facilitate stress tests to ensure it is operable. Countries should develop their own national plans seeking coherence and compatibility with the EU plan. The scope of public health risk assessments will be broadened to support an all-hazards approach. Consequently, depending on the type of health threat, one or several EU agencies may contribute to the risk assessment.

An important new aspect included in the Regulation on serious-cross border threats to health is the possibility to declare a public health emergency at EU level. Such declaration will provide the basis to identify and ensure the availability of relevant critical medicines and coordinate associated measures to address the public health emergency [4], flexible mechanisms to develop, procure, manage and deploy medical countermeasures [5], as well as the activation of support from the ECDC to mobilise and deploy the ‘EU Health Task Force’ [3].

Any future path for health must be taken together with global partners. The European Global Health strategy [12] emphasises the role of the EU in global health, with a One Health and a ‘health in all policies’ approach at its very core. The EU will support strong multilateral systems around the World Health Organization. It actively participates in the negotiation of the future pandemic agreement and in the revision of the International Health Regulations, and it will engage in the Pandemic fund hosted by the World Bank. To improve equity in the access to vaccines and other countermeasures, the EU will boost local manufacturing capacities and strengthen health systems.

Further pillars of the European Health Union are Europe’s Beating Cancer Plan that aims to improve cancer detection and treatment, to ensure better integrated and comprehensive cancer care and to address unequal access to quality cancer care and medicines [6], as well as the Pharmaceutical Strategy [7]. The latter, adopted on 26 November 2020, aims to give all Europeans equal access to affordable, safe and effective medicines and treatments. It will drive innovation in unmet medical needs and help secure access to affordable, high quality, safe and greener medicines in the EU. The Pharmaceutical Strategy should also boost EU resilience by promoting global and diversified supply chains. To secure supply in the EU, it includes stronger obligations for supply, transparency and earlier notification of shortages. The strategy modernises the existing regulatory framework and supports research and technologies that can be applied in healthcare. It addresses several antimicrobial resistance challenges, including the lack of investment in antimicrobials and the inappropriate use of antibiotics, and also covers actions on improving healthcare professionals’ and European citizens’ awareness on antimicrobial resistance.

The COVID-19 pandemic has uncovered the need for improvement in the digital realm. However, there has been much progress with accelerated development and use of digital tools and electronic health records. To further harness the benefits of digital tools to inform health policy, the EC is building the European Health Data Space (EHDS) [8]. The EHDS is both a health-specific data sharing framework establishing clear rules, common standards and practices, and a governance framework. By offering a consistent, secure, trustworthy and efficient framework for the use of health data, it will allow individuals to have digital access to and control of their personal health data. It will support their free movement by ensuring that health data follows them. Furthermore, EHDS will enable the use of electronic health data for research, innovation, policy making, patient safety, statistics and regulatory purposes.

A strong European Health Union can only survive and thrive with committed investment. The EU4Health programme will add €5.3 billion in health promotion, diagnosis and treatment, and care to help countries boost their health systems, strengthen their healthcare workforce, invest in trainings and advance their digital transformation.

Multi-sectoral and cross-border coordination are needed to effectively face the health threats that may lie ahead. The new European Health Union will respond to this need. Its complementary pillars support EU countries in preparing for and responding to European and global health crises. Working together to improve prevention and treatment of diseases and guarantee availability of affordable and innovative medical supplies is of major importance. With a stronger European Health Union, we should be better prepared to face any future health threat with confidence.

## Note

Sandra Gallina is the Director General of the Directorate-General for Health and Food Safety (DG SANTE).
